# Ultrasound-guided peripheral abdominal wall blocks

**DOI:** 10.6061/clinics/2021/e2170

**Published:** 2021-01-11

**Authors:** Hermann dos Santos Fernandes, Artur Salgado de Azevedo, Thiago Camargo Ferreira, Shirley Andrade Santos, Joel Avancini Rocha-Filho, Joaquim Edson Vieira

**Affiliations:** Divisao de Anestesiologia, Hospital das Clinicas HCFMUSP, Faculdade de Medicina, Universidade de Sao Paulo, Sao Paulo, SP, BR

**Keywords:** Regional Anesthesia, Peripheral Nerve, Analgesia, Ultrasonography, Abdominal Wall

## Abstract

The practice of regional anesthesia is in a state of progressive evolution, mainly due to the advent of ultrasound as an anesthesiologist's instrument. Alternative techniques for postoperative analgesia of abdominal surgeries, such as transversus abdominis plane block, oblique subcostal transversus abdominis plane block, rectus abdominis muscle sheath block, ilioinguinal and iliohypogastric nerve block, and quadratus lumborum plane block, have proven useful, with good analgesic efficacy, especially when neuroaxial techniques (spinal anesthesia or epidural anesthesia) are not possible. This review discusses such blockades in detail, including the anatomical principles, indications, techniques, and potential complications.

## INTRODUCTION

Open abdominal surgeries are often associated with severe postoperative pain. The use of an epidural approach (epidural anesthesia / analgesia, with or without an epidural catheter) remains the gold standard for analgesia after abdominal surgery. However, neuroaxial methods (epidural or intrathecal) are not possible in some patients, due to use of anticoagulant agents, coagulopathy, hemodynamic instability, and other contraindications to epidural space access (1). On these occasions, alternative modes of analgesia are necessary, and peripheral techniques are reliable and beneficial.

**Figure 1:**
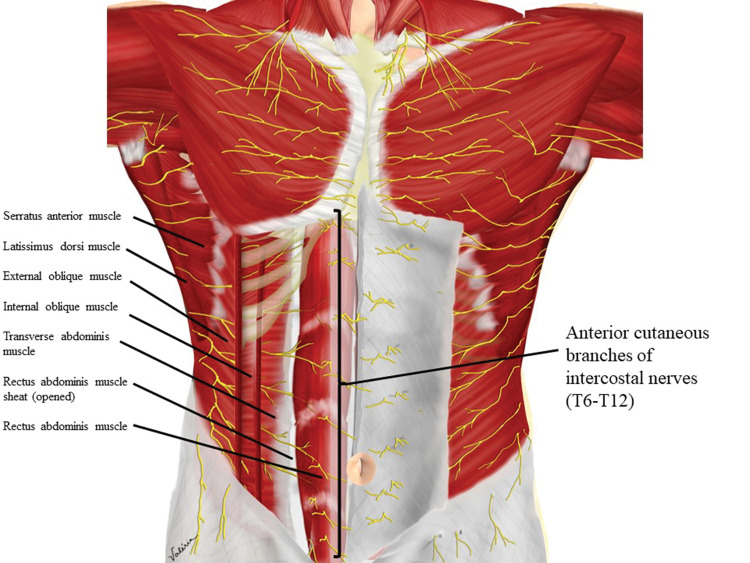
Innervation of the anterolateral abdominal wall.

The anterolateral abdominal wall is innervated by thoracolumbar nerves T6 to L1, through extensions of the intercostal, subcostal, ilioguinal, and iliohypogastric nerves. Branches of these nerves reach the anterior and lateral portions of the abdomen, passing through the intermuscular fascial planes, between the internal oblique (IO) and transverse abdominis (TA) muscles, reaching the midline after perforating the rectus abdominis muscle sheath (RAS) (Figure 1) (1,2). This anatomical feature allows a local anesthetic to be injected into an intermuscular plane and its dispersion to reach the desired nerves. Ultrasound (US)-guided injection into an intermuscular plane is technically easier to perform than identifying specific peripheral nerves. This approach provides easy and versatile peripheral block techniques (3).

In this article, we will describe the most useful US-guided peripheral blocks for abdominal wall analgesia.

## TRANSVERSUS ABDOMINIS PLANE (TAP) BLOCK

First described based on anatomical markers by Dr. Rafi (4), the advent of US popularized the technique for its added safety.

### Anatomy

**Figure 2:**
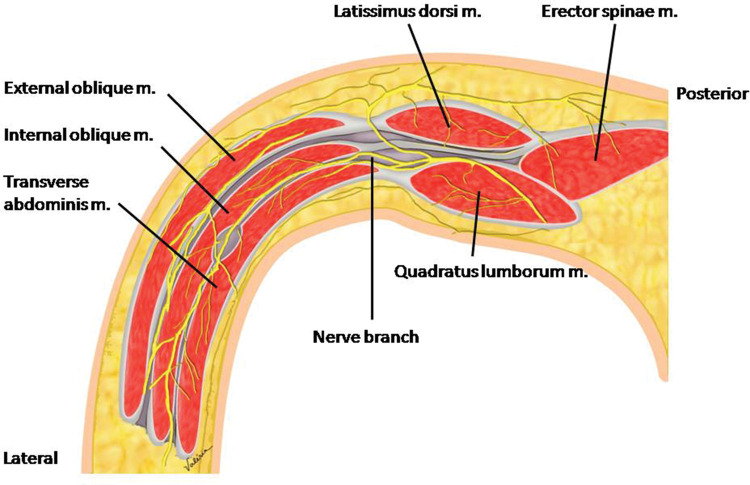
Cross section showing the inter-muscular plane between the IO and TA muscles with the nerve branches to be blocked.

The anterior branches of intercostal nerves T7 to T12 and the anterior branch of the spinal nerve L1 are responsible for innervation of the lower anterolateral abdominal wall and are arranged in the plane between the IO muscle and the TA muscle (Figures 1 and 2) (3,5).

### Indications

Analgesia for lower abdominal and infra-umbilical surgeries: Pfannenstiel incision, abdominal hysterectomy, cesarean section, herniorrhaphy, correction of orchidopexy, appendectomy, cystostomy, lower middle laparotomies, and retropubic prostatectomy (6-9). It has shown inferior analgesia compared to intrathecal morphine, although it has fewer adverse effects (10).

### Technique

**Figure 3:**
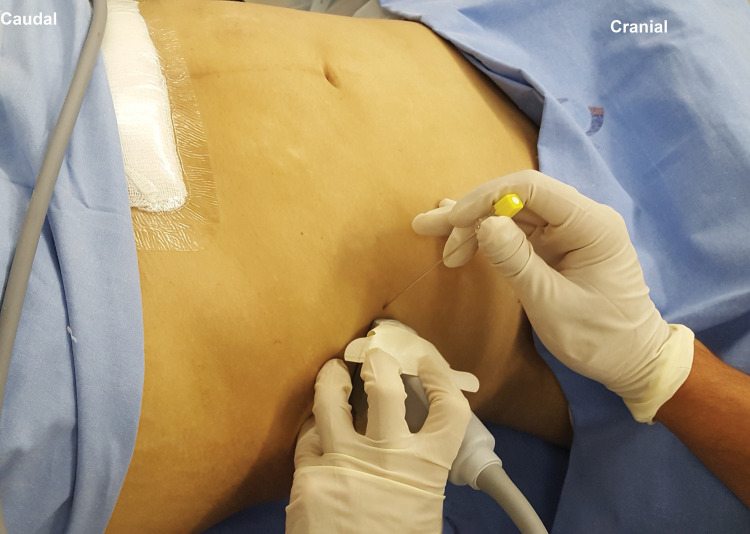
Probe and needle position to perform TAP block.

**Figure 4:**
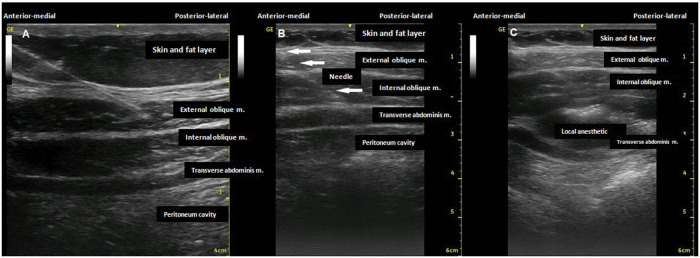
A. Muscle planes visualized during TAP block execution. B. Needle with tip located between IO and TA muscles, C. Local anesthetic spread in the plane between IO and TA muscles.

With the patient in a dorsal or lateral decubitus position (11), a linear US probe (10-18 MHz), with a programmed depth of 3-5cm (depending on the thickness of the fat layer), should be positioned transversely at the midpoint between the lower costal margin and the iliac crest, right on the anterior axillary line (Figure 3). Under US view, three well-defined muscle planes can be identified below the skin and subcutaneous tissue: external oblique muscle (EO), IO muscle, and TA muscle. The IO muscle is usually the most prominent of the three. Below the third muscle plane, moving structures (viscera with peristalsis, respiratory movement within the abdominal cavity) are noted (Figure 4A). If it is difficult to visualize the three muscle planes, the probe should be slid to the midline, and the rectus abdominis (RA) muscle will be easily identified as a fusiform muscle surrounded by its sheath. By slowly sliding the probe in a lateral direction from the RA, in its lateral border (where the linea semilunaris is formed), the medial edge of the OI muscle is visualized. When good visualization of the three muscle planes is obtained, a 20-22G needle, 90-100 mm in length, should be introduced in-plane from medial to lateral, aiming to place its tip in the fascial plane between the IO and TA muscles (Figure 4B). To facilitate needle visualization, it is possible to inject saline fractions (1-2 mL) for local hydrodissection. With the needle in place, 15-20 mL of local anesthetic (Ropivacaine 0.25%-0.5%, Bupivacaine 0.25%-0.5%, Levobupivacaine 0.25%-0.5%) should be injected after negative aspiration for blood or other materials. The correct injection site is confirmed by visualization of the spread of hypoechoic fluid between the OI and TA muscles (Figure 4C). It should be performed bilaterally for midline incisions.

### Complications

Possible complications include peritoneal perforation, visceral injury, intraperitoneal injection, local anesthetic systemic toxicity, and hematoma. However, the use of US considerably reduces the incidence of complications (12).

## OBLIQUE SUBCOSTAL TRANSVERSUS ABDOMINIS PLANE BLOCK (OSTAP)

Also known as high TAP block, OSTAP was first described by Hebbard in 2008, who suggested its use for abdominal surgeries with supra-umbilical incisions (13).

### Anatomy

The upper abdomen is innervated by the terminal branches of the T6-T9 spinal nerves, which run in the neurovascular plane between the IO and TA muscles, more medially than the branches responsible for the inferior wall innervation, in relation to the anterior axillary line (Figure 1) (2,5).

### Indications

Upper abdominal surgeries, such as open cholecystectomies. Its bilateral application may also provide analgesia for supra-umbilical median incisions (2,13,14). The use of traditional TAP block and concomitant OSTAP may be called double TAP and provides analgesia for both the lower and upper abdomen. Bilateral double TAP block is called a four-quadrant TAP block and provides analgesia for the entire anterior abdominal wall and may be indicated for median laparotomies in cases in which the neuroaxis cannot be used (2,15,16).

### Technique

**Figure 5:**
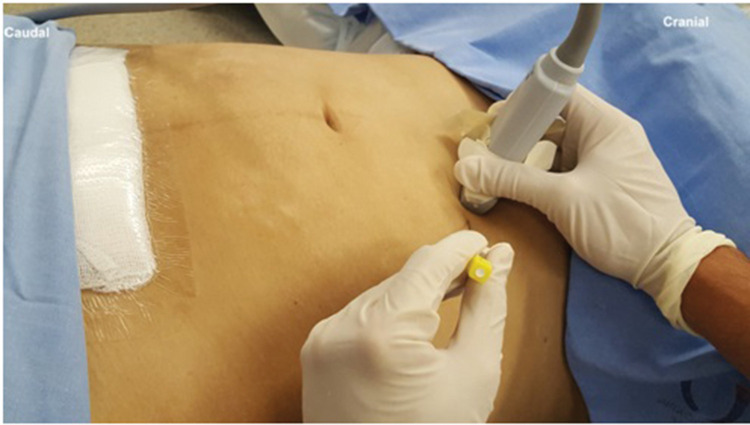
Probe and needle position to perform OSTAP block.

**Figure 6:**
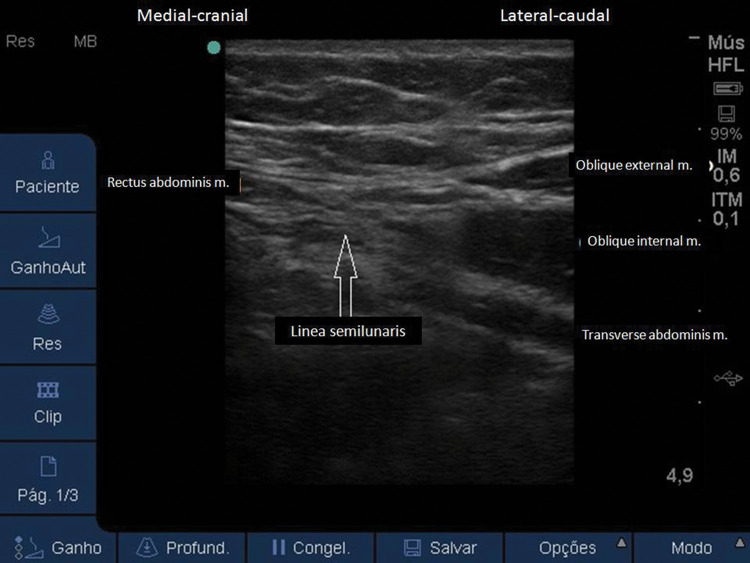
Ultrasound image with visualization of the lateral portion of the RA muscle, linea semilunaris and abdominal muscles planes laterally.

**Figure 7:**
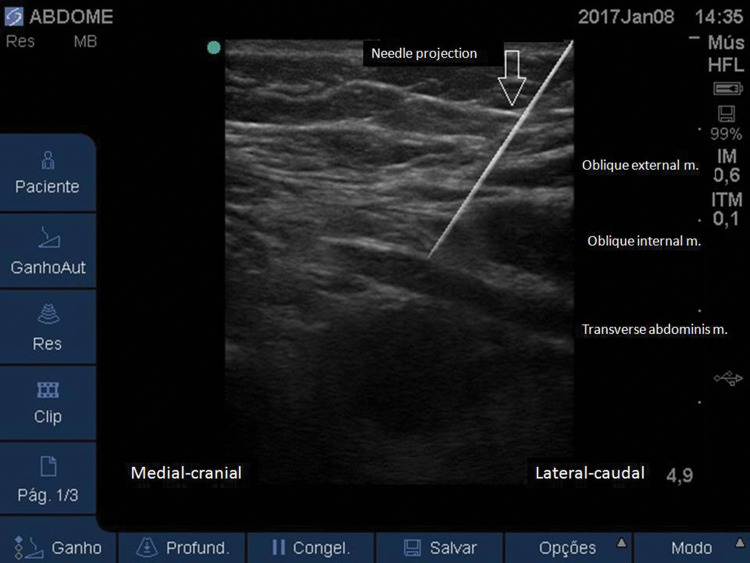
Ultrasound image showing the proper position of the needle tip in the plane between the IO and TA muscles.

Similar to the traditional TAP block, with the patient in a supine position, a linear US probe (10-18 MHz) should be positioned at the inferior border of the costal cage, at the point it crosses the hemiclavicular line (Figure 5), in an oblique position. In this location, the lateral border of the RA muscle (with fusiform image) and its sheath can be visualized (Figure 6). Lateral to the RA muscle, the three planes of the EO, IO, and TA muscles can be visualized (Figure 6). With this ultrasound image, a 20-22G, 50-100-mm-long needle should be introduced in-plane from lateral to medial and its tip directed to the fascial plane between the OI and TA muscles, very close to the linea semilunaris (lateral edge of the RA sheath) (Figure 7). With the needle in place, 20 to 25 mL of local anesthetic (Ropivacaine 0.25%-0.5%, Bupivacaine 0.25%-0.5%, Levobupivacaine 0.25%-0.5%) should be injected after negative aspiration for blood or other materials. The correct injection site is confirmed by visualization of hypoechoic fluid among IO and TA muscles, and posterior sheath of the RA muscle (2,3).

### Complications

Similar to TAP block, possible complications are peritoneal perforation, visceral injury, and intra-peritoneal injection. The use of US carries lower risks (12).

## RECTUS ABDOMINIS MUSCLE SHEATH (RAS) BLOCK

Initially described in 1899, RAS block was used for abdominal wall relaxation during laparotomies prior to the introduction of neuromuscular blocking agents.

### Anatomy

The terminal branches of the T7-T11 intercostal nerves run between the IO and TA muscles toward the anterior portions of the abdominal wall, penetrating the posterior leaflet of the RAS to reach the midline (Figure 1) (17).

### Indications

Analgesia for midline umbilical and incisional hernia surgeries, median incisions, and median longitudinal laparotomies (17-21).

### Technique

**Figure 8:**
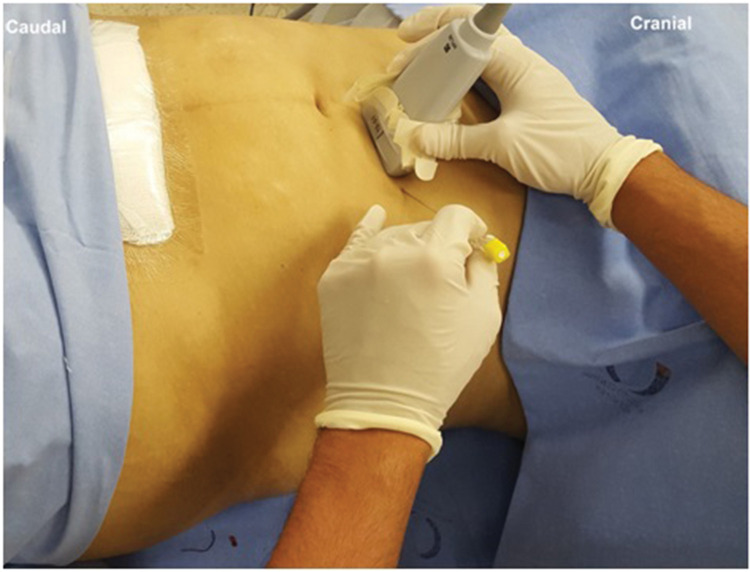
Probe and needle position for performing RAS block.

**Figure 9:**
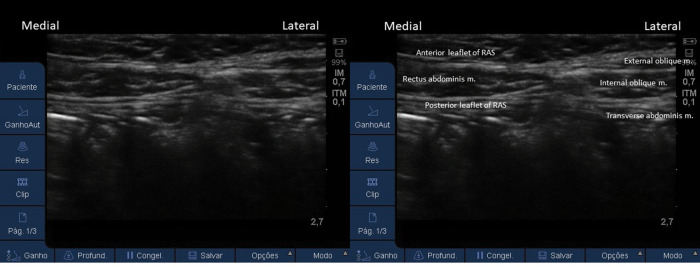
Ultrasound image of the RA muscle enveloped by its sheath and the plane muscles, laterally.

**Figure 10:**
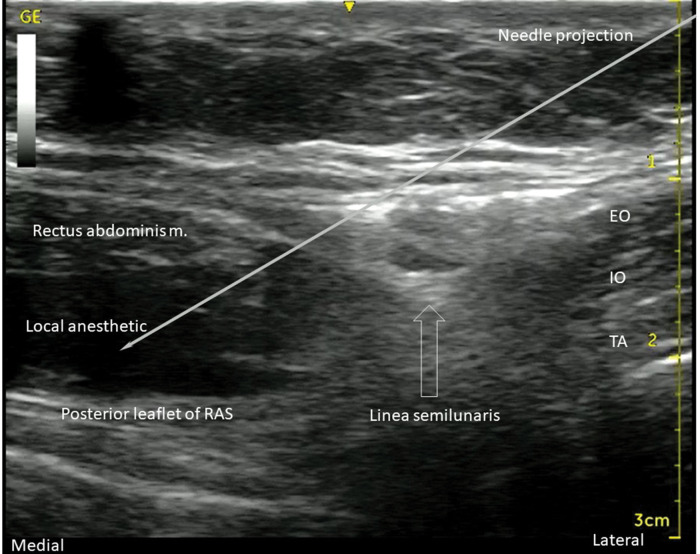
US image, with the projection of the needle position, and the local anesthetic spread between the RA muscle and posterior leaflet of RAS. EO: external oblique muscle; IO: internal oblique muscle; TA: transverse abdominis muscle.

With the patient in a supine position, a linear US probe (10-18 MHz) should be positioned transversely, approximately 3 cm above the umbilical scar over the RA muscle (Figure 8), which is easily identified by US by its fusiform shape and surrounding sheath (Figure 9). Maintaining the image of the RA muscle and its most lateral portion on US, a 20-22G, 50-100-mm-long needle should be introduced in-plane from lateral to medial, aiming its tip between the RA muscle and the posterior portion of the RAS (Figure 10), inside the RAS. With the needle in the correct place, 10-15 mL of local anesthetic (Ropivacaine 0.25%-0.5%, Bupivacaine 0.25%-0.5%, or Levobupivacaine 0.25%-0.5%) should be injected after negative aspiration for blood or other materials. The correct injection site is confirmed by visualization of the spread of hypoechoic fluid between the RA muscle and the posterior leaflet of the RAS (Figure 10). For further confirmation of anesthetic injection at the correct site, it is possible to slide the US cranially and caudally and check for local anesthetic spread along to the RAS (1,3). It must be performed bilaterally.

### Complications

Surgical wound infection and accidental intra-peritoneal puncture are complications already reported in the execution of this block (3).

## ILIOINGUINAL (II) AND ILIOHYPOGASTRIC (IH) NERVE BLOCK

The II and IH nerve block techniques have been used for analgesia of infra-umbilical and inguinal surgical procedures, especially in children, and their analgesic effect is comparable to that provided by caudal epidural block (22-24).

### Anatomy

**Figure 11:**
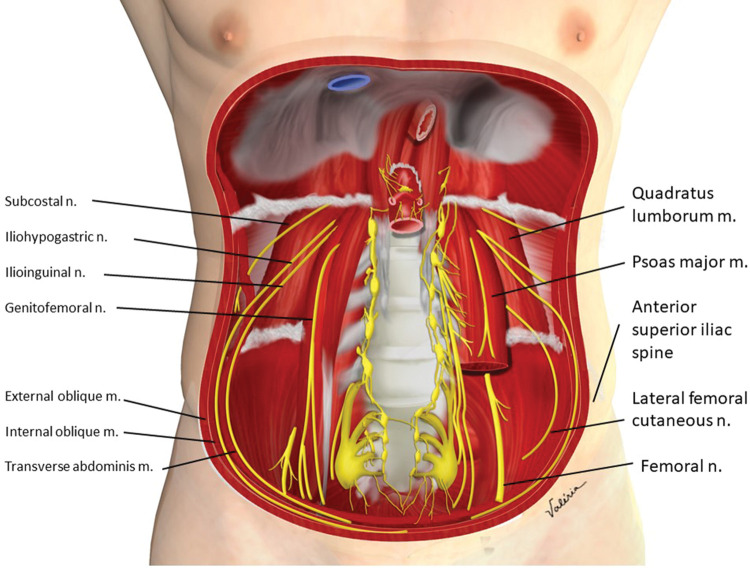
Arrangement and path of the II and IH nerves in the abdominal wall.

The II and IH nerves emerge from the first lumbar spinal nerve, pass through the superior and lateral portion of the Psoas Major (PM) muscle, projecting to the inferior anterolateral abdominal wall, passing, at the level of the anterior superior iliac spine, between the IO and TA muscles (Figures 1 and 11) (3).

### Indications

The main indications for II and IH nerve block are analgesia for inguinal hernia, orchidopexy, varicocele surgery, hydrocele surgery, open appendectomy, and obstetric and gynecological surgeries (22-30).

### Technique

**Figure 12:**
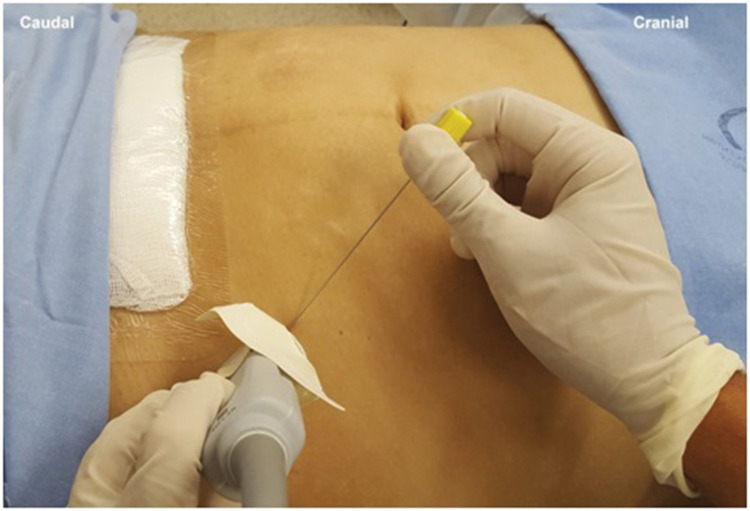
Probe and needle position for II and IH nerve block.

**Figure 13:**
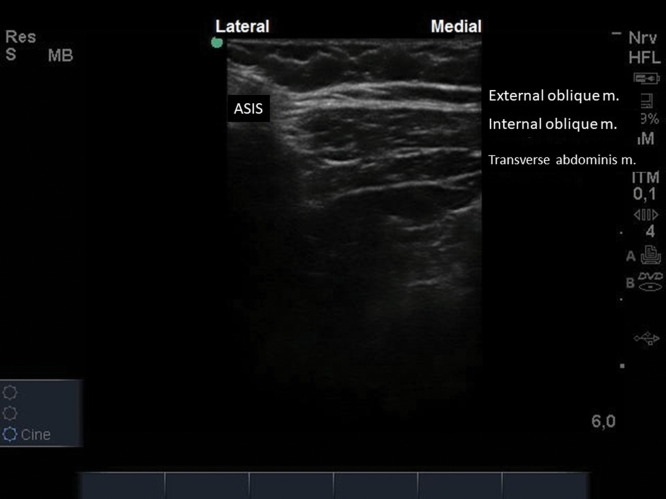
ASIS (anterior superior iliac spine) laterally, and muscles of the abdominal wall medially.

**Figure 14:**
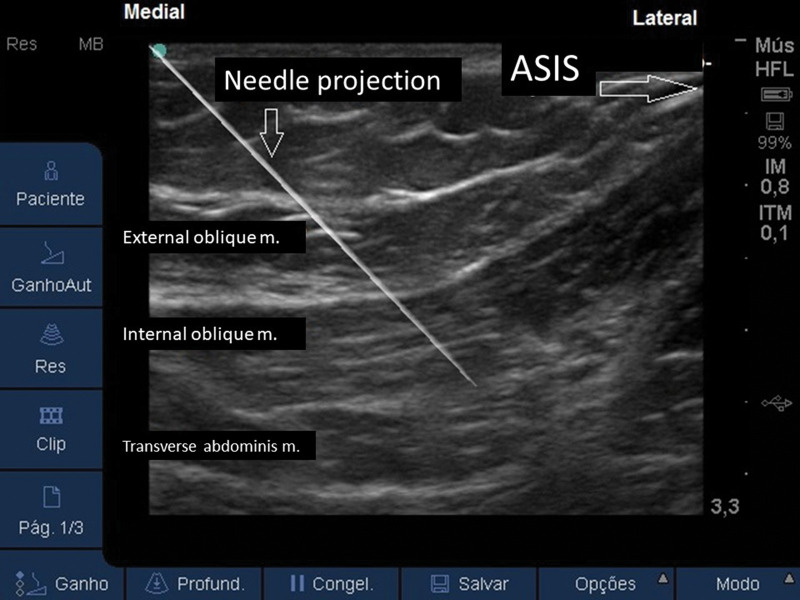
US image, with the projection needle, with the tip located in the plane between the IO and TA muscles. ASIS: anterior superior iliac spine.

With the patient in a supine position, a linear US probe (10-18 MHz) should be positioned obliquely over an imaginary line from the anterior superior iliac spine to the umbilical scar (Figure 12). In this position, the US image demonstrates the anterior superior iliac spine (bony surface with posterior acoustic shadow) laterally, and the medial muscle planes of the EO, IO, and TA muscles (Figure 13). Maintaining this image, a 20-22G, 50-100-mm-long needle should be introduced in-plane from medial to lateral to place its tip in the plane between the OI and TA muscles (Figure 14). With the needle in place, 10 to 15 mL of local anesthetic (Ropivacaine 0.25%-0.5%, Bupivacaine 0.25%-0.5%, or Levobupivacaine 0.25%-0.5%) should be injected after negative aspiration for blood or other materials. The correct injection site is confirmed by visualization of the spread of hypo-echoic fluid between the IO and TA muscles (1,3).

### Complications

Accidental intra-peritoneal puncture and femoral nerve block are evidenced as complications (22-30).

## QUADRATUS LUMBORUM (QL) BLOCK

Recently described by Blanco and modified by Sauter et al., the quadratus lumborum fascial block (QLB) is considered an extension of the TAP block (3). A local anesthetic is injected adjacent to the QL muscle, aiming to anesthetize the thoracolumbar nerves.

### Anatomy

The QL muscle is a quadrangular-shaped posterior abdominal wall muscle that inserts inferiorly into the iliac crest, cranially in the 12th rib, medially in the transverse processes from L1 to L4, and has a free lateral border. On its anterior face, the QL muscle is related to the PM muscle and on its posterior face, to the spinal erector muscle group. The muscle is surrounded by the thoracolumbar fascia (Figure 11) (31).

### Indications

Many case reports and randomized trials include, as indications, analgesia for gynecological and lower abdominal surgery, Pfannenstiel incision for cesarean sections, proctosigmoidectomy, hip surgery, abdominal hernioplasty, nephrectomy, and laparotomy. There are several case reports with different indications of QLB for sensory block between T7 and L2 (31). Higher coverage for regions above the umbilical scar is reported compared with TAP block (32) in addition to a possible action on visceral pain, due to the spread of local anesthetic through the thoracolumbar fascia layers, reaching higher and proximal levels towards thoracic and lumbar nerve roots, reaching the paravertebral space, and blocking autonomic pathways (33).

### Technique

Three techniques for QLB have been described and named according to the position of the needle tip in relation to the QL muscle: QLB type 1, or lateral; type 2, or posterior; and trans-muscular, or anterior (31).

**Figure 15:**
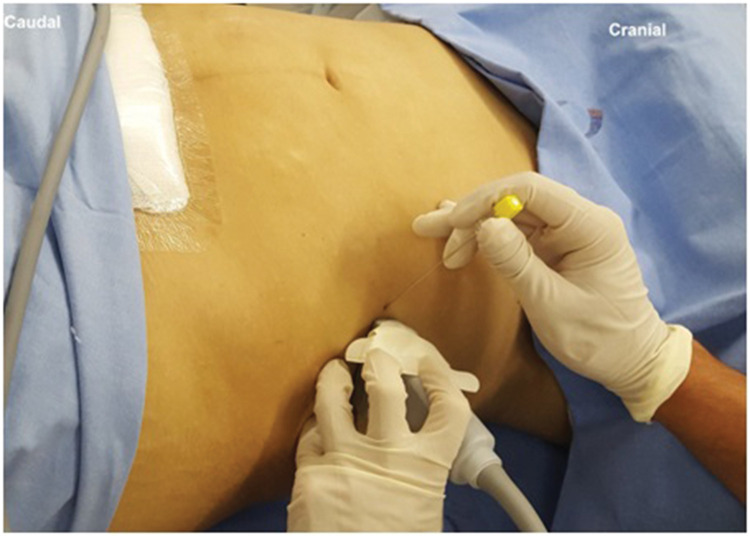
US probe and needle position for QL blocks types 1 (lateral) and 2 (posterior).

**Figure 16:**
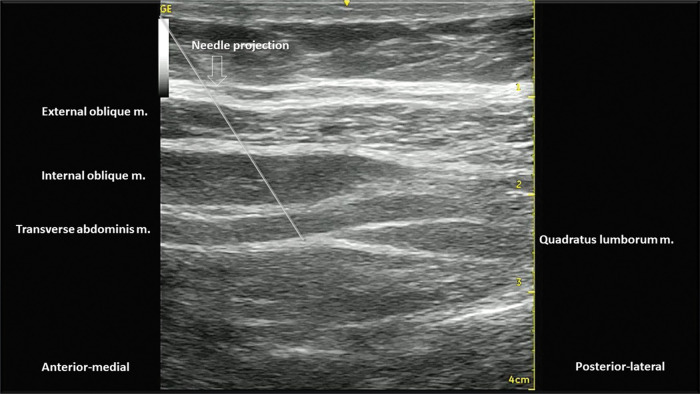
US image with needle position projection. The tip is located deeper than the TA muscle, laterally to the lateral border of the QL muscle.

**Figure 17:**
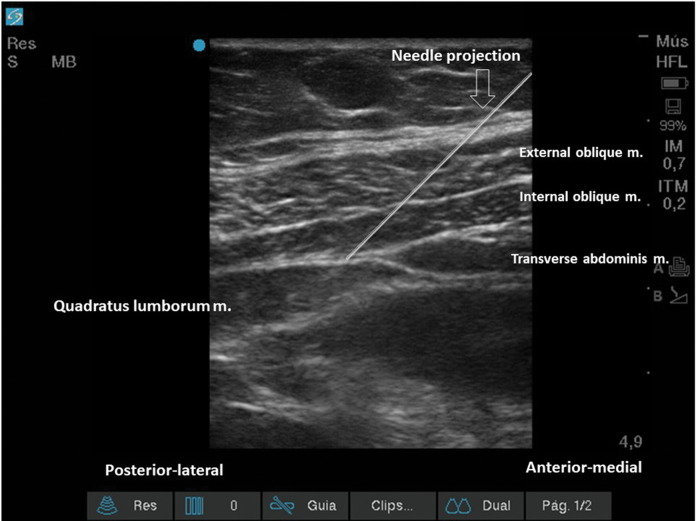
US image, with needle projection. Its tip is located on the posterior-lateral border of the TA muscle, posterior to the QL muscle.

**Figure 18:**
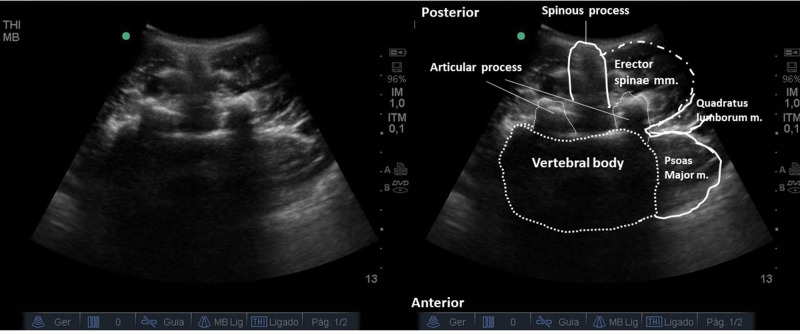
US image of the transducer on the posterior midline in the transverse position. Spine vertebrae and important paravertebral structures for trans-muscular QL block.

**Figure 19:**
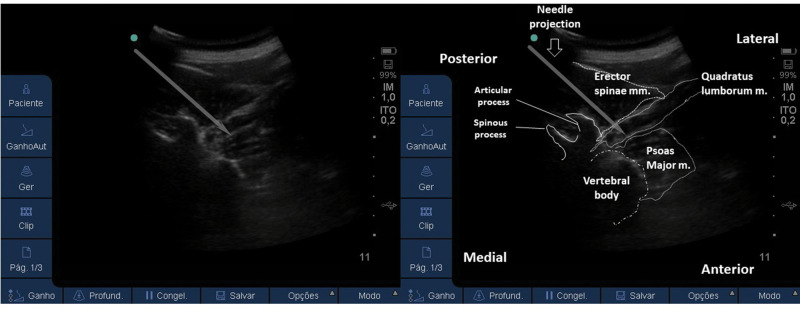
US image of the transducer in the lateral position, medially tilted, with a view of the lateral aspect of the vertebrae. To perform the block, the needle may be advanced from posterior to anterior (as the above image) or from lateral to medial. Its tip must cross the QL muscle and be located in the plane between the QL and PM muscles.

1. Lateral QLB (Type 1):
  With the patient in a supine or partial lateral position, a high- or low-frequency transducer is positioned transversely 2 cm above the umbilicus and then slid laterally, until the lateral border of the QL muscle is seen, deeper than the TA muscle (Figures 15 and 16). A 100-150 mm, 20-22G needle is inserted in-plane, anterior to posterior orientation, and the local anesthetic is deposited on the anterior-lateral border of the QL muscle, at its junction with the fascia transversalis (Figure 16). With the needle in the correct place, 20-25 mL of local anesthetic (Ropivacaine 0.25%-0.5%, Bupivacaine 0.25%-0.5%, or Levobupivacaine 0.25%-0.5%) must be injected, after negative aspiration for blood or other materials. This approach has the advantage of being technically easier to perform, especially for professionals with expertise in TAP block.
2. Posterior QLB (Type 2):
  With the patient in a supine or partial lateral position, a high- or low-frequency transducer is positioned transversely 2 cm above the umbilicus and then slid laterally, until the lateral border of the QL muscle is seen, deeper than the TA muscle (Figures 15 and 16). From that point, the probe must be slid even more posterior-laterally, until it reaches the lateral-posterior border of the TA muscle, deeper than the IO muscle (Figure 17). A 100-150 mm, 20-22G needle should be inserted in-plane, from the anterior-medial to the lateral-posterior direction, and its tip located between the QL and IO muscles. Twenty to twenty-five milliliters of local anesthetic (Ropivacaine 0.25%-0.5%, Bupivacaine 0.25%-0.5%, or Levobupivacaine 0.25%-0.5%) should be injected after negative aspiration for blood or other materials. This approach has the advantage of better ultrasound resolution and a lower risk of complications because it is more superficial.
3. Anterior or trans-muscular QLB:
  With the patient in a ventral or lateral position, a low-frequency curvilinear transducer may be positioned, in transverse orientation, on the lumbar midline, at the L2 level, or above the iliac crest level. The US view must include the posterior vertebrae and the paravertebral structures (Figure 18). Next, the transducer must be slid to the side to be blocked and tilted medially. It is possible to view the lateral aspect of the vertebrae (“killer whale image”) and paravertebral muscles (erector spinae muscle, QL muscle, and PM muscle) (Figure 19). A 100-150 mm, 20-22G needle should be inserted, in-plane, from posterior to anterior or from lateral to medial, and the tip must be located in the plane between the QL and PM muscles. A volume of 20-25 mL of local anesthetic (Ropivacaine 0.25%-0.5%, Bupivacaine 0.25%-0.5%, or Levobupivacaine 0.25-0.5%) is injected, and a hypoechoic spread is observed between the QL and PM muscles. This technique can be considered more laborious due to the patient's positioning and because it is deeper. It is more difficult to obtain adequate ultrasound imaging, and there is a higher risk of complications (3,31,34). This technique may be considered very similar (if not identical) to the posterior lumbar plexus block (35).

### Complications

Due to local anesthetic spread to the lumbar plexus, motor block or lower limb weakness may occur. In addition, hypotension may occur due to the spreading of local anesthetic to the paravertebral or epidural spaces. Complications related to needle trauma include inadvertent renal puncture, retroperitoneal hematoma, peritoneal perforation, viscera injury, and intra-peritoneal injection (3).

## CONCLUSIONS

Neuroaxial methods of postoperative analgesia for abdominal surgeries are still the best evidenced options (continuous epidural through epidural catheter and intrathecal anesthesia with long-term opioid injection). However, these routes are not always available. In these cases, there are several options for regional anesthesia with the property of providing satisfactory postoperative analgesia for abdominal surgeries. The blocks described were already used in regional anesthesia, even before the advent of US, by techniques based on anatomical surface markers and fascial sensations of clicks. However, practice and evidence suggest that US-guided regional anesthesia techniques are safer and more effective, allowing greater use of specific blocks with their benefits when epidural or spinal anesthesia are not viable options.

## AUTHOR CONTRIBUTIONS

Fernandes HS was responsible for the literature review, pictures production, and manuscript writing. Azevedo AS, Ferreira TC and Santos SA were responsible for the literature review, and manuscript writing. Rocha-Filho JA and Vieira JE were responsible for the literature review, and manuscript corrections and review.
